# Isolation and Abiotic Stress Resistance Analyses of a Catalase Gene from* Ipomoea batatas* (L.) Lam

**DOI:** 10.1155/2017/6847532

**Published:** 2017-05-30

**Authors:** Bin Yong, Xiaoyan Wang, Pan Xu, Haiyan Zheng, Xueting Fei, Zixi Hong, Qinqin Ma, Yuzhi Miao, Xianghua Yuan, Yusong Jiang, Huanhuan Shao

**Affiliations:** ^1^College of Life Sciences, Sichuan Normal University, Chengdu, Sichuan 610101, China; ^2^College of Life Science & Forestry, Chongqing University of Art & Science, Yongchuan, Chongqing 402160, China

## Abstract

As an indicator of the antioxidant capability of plants, catalase can detoxify reactive oxygen species (ROS) generated by environmental stresses. Sweet potato is one of the top six most important crops in the world. However, its catalases remain largely unknown. In this study, a catalase encoding gene,* IbCAT2 *(accession number: KY615708), was identified and cloned from sweet potato cv. Xushu 18. It contained a 1479 nucleotides' open reading frame (ORF). S-R-L, Q-K-L, and a putative calmodulin binding domain were located at the C-terminus of IbCAT2, which suggests that IbCAT2 could be a peroxisomal catalase. Next-generation sequencing (NGS) based quantitative analyses showed that* IbCAT2* was mainly expressed in young leaves and expanding tuberous roots under normal conditions. When exposed to 10% PEG6000 or 200 mmol/L NaCl solutions,* IbCAT2* was upregulated rapidly in the first 11 days and then downregulated, although different tissues showed different degree of change. Overexpression of* IbCAT2* conferred salt and drought tolerance in* Escherichia coli *and* Saccharomyces cerevisiae*. The positive response of* IbCAT2* to abiotic stresses suggested that* IbCAT2* might play an important role in stress responses.

## 1. Introduction

Root and tuber crops are basic components of human diets in the tropics and subtropics. Among these crops, sweet potato [*Ipomoea batatas* L. (Lam.)] is widely grown in all tropical and subtropical areas of the world because of its rich nutrient density, low input requirement, and wide range of uses [1–3]. It can produce more biomass per unit area per unit time than any other food crop [3]. About 130 million metric tons of tuberous roots are produced worldwide each year, most of which are grown in China [3]. Moreover, sweet potato can be grown in marginal conditions better than most other crops, meaning its growth does not compete for land with other food crops. Several groups have shown interest in the sweet potato because it is a genetically challenging hexaploid plant (2*n* = 6*x* = 90) with a genome size between 2,200 and 3,000 Mbp [4, 5]. Most recently, growing attention to the health benefits attributed to sweet potato has stimulated a renewed interest in this crop [6].

Since plants lack mobility they cannot avoid exposure to environmental stresses. As such, they have evolved several important strategies to adapt to these biotic and abiotic stresses, including stress escape, stress avoidance, and stress tolerance [7]. Most stress responses in plants involve the accumulation of reactive oxygen species (ROS) and ROS-induced damage of proteins, DNA, and lipids [8–10]. In plants, about 1% of the total O_2_ is consumed to produce ROS [11]. One ROS species, hydrogen peroxide (H_2_O_2_), has received particular attention as an important signaling molecule involved in plant development and stress responses benefit from its relative stability. H_2_O_2_ is generated by a two-electron reduction of O_2_, catalyzed by certain oxidases or indirectly via reduction or dismutation of O_2_^−^ [12–14]. Accumulated H_2_O_2_ can be then metabolized by catalases (CATs), ascorbate peroxidases (APX), peroxiredoxins (PRX), glutathione/thioredoxin peroxidases (GPX), and glutathione S-transferases (GST) [15–19]. CATs catalyze a dismutation reaction to convert two molecules of H_2_O_2_ to H_2_O and O_2_, which distinguish them from other H_2_O_2_-metabolizing enzymes in that they do not require a reductant [20]. A large number of studies have revealed CAT to be a sink for H_2_O_2_ and indispensable for stress defense in plants [21–23]. Angiosperm species usually contain three catalase genes [15, 24, 25].

In sweet potato, only one full-length catalase encoding gene* (SPCAT1)* has previously been cloned and characterized [26–28]. In this study, another catalase encoding gene (*IbCAT2*) was identified from the transcriptome of sweet potato. The spatiotemporal expression patterns and putative function of* IbCAT2* were thoroughly characterized.

## 2. Materials and Methods

### 2.1. Plant Materials

Stem cuts of sweet potato (*I. batatas* cv. Xushu 18) were planted in 30 cm plastic pots in the glasshouse of Sichuan Academy of Agricultural Sciences under natural irradiance (irrigated with 500 mL water every three days) in Chengdu, Sichuan Province of China from May 10 to October 10. Tissues for experiments were sampled at 9:00 AM three months after planting and immediately snap-frozen in nitrogen prior to further processing. For stress analysis, stem cuts of sweet potato were planted in the glasshouse for three months under natural irradiance. They were then divided into three groups: a well-watered group as control, a salt treatment group, and a drought treatment group. Each group contained >50 plants. The salt and drought treatment groups were irrigated with 200 mmol/L NaCl solution (500 mL) and 10% PEG6000 solution (500 mL), respectively, every three days for 30 days, while the control group was irrigated with an equal volume of water every three days for 30 days. All tissue samples were collected at 9:00 AM.

### 2.2. RNA Extraction and Sequence Cloning

Total RNAs were isolated from sweet potato samples using TRIzol reagent (Invitrogen, USA) according to the manufacturer's instructions. RNA quality and purity were assessed by agarose gel electrophoresis. The total RNAs were then reversely transcribed with PrimeScriptTMRT Reagent Kit (TaKaRa, Japan) using Oligo(dT) as primer. Cloning primers were designed according to the assembled contig of* IbCAT2* in the sweet potato transcriptome [32, 34] using Primer Premier 5.0 (PREMIER Biosoft International, CA, USA). Polymerase Chain Reaction (PCR) was performed using KOD-Plus-Neo (TOYOBO, Japan) with IbCAT F1 (5′-GATATCATGGATCCTTATCAGCACCG-3′) and IbCAT R1 (5′-GGAATTCTCACATTGTTGGCCGCAC-3′; bp position: 1049–1069) as primers with 35 cycles of 2 min at 94°C, 10 s at 98°C, 30 s at 56°C, and 45 s at 68°C. The PCR product was separated by 1% agarose gel and purified by DNA gel extraction kit (OMEGA, USA). It was then double digested by* Eco*R I and* Eco*R V (Fermentas, USA). The pET-32a(+) was also double digested by* Eco*R I and* Eco*R V (Fermentas, USA). Restricted DNA products were then separated by 1% agarose gel, purified by DNA gel extraction kit (OMEGA, USA), and then ligated with pET-32a(+) by T4 DNA ligase (TaKaRa, Japan). The recombinant plasmid was transformed into the* Escherichia coli* host strain JM109. Positive clones were sequenced with an ABI 3730 instrument.

### 2.3. RNA-Seq Based Expression Analyses

Next-generation sequencing (NGS) based digital gene expression (DGE) profiling is a tag sequencing method for global gene expression quantification. In previous study, seven different sweet potato tissues were collected for DGE analysis [32]. In this study, 21 bp DGE tags of these seven different tissues were retrieved from the NCBI's Sequence Read Archive database (http://www.ncbi.nlm.nih.gov/Traces/sra) and aligned to* IbCAT2*. The number of mapped tags was counted according to the mapping results for each sample and used to quantify expression using the TPM algorithm (transcripts per million clean tags) [35, 36].

### 2.4. Quantitative Real-Time Polymerase Chain Reaction (qRT-PCR)

Young leaves (YL), stems (ST), and expanding tuberous roots (ETR), with or without stress treatment, were collected at 0, 2, 11, and 20 days after stress treatment. Each tissue sample was collected and pooled together from five plants and immediately snap-frozen in nitrogen prior to further processing. For each time point, three biological replicates were collected. Total RNAs were extracted from each sample using TRIzol reagent (Invitrogen, USA) and genomic DNA was digested by DNase I (Fermentas, USA) according to the manufacturer's instructions. RNA quality and purity were assessed using the OD_260/230_ ratio and RNA integrity number (RIN) with the SMA3000 and the Agilent 2100 Bioanalyzer, respectively. Equal amounts of qualified total RNAs (500 ng RNAs) from each tissue sample were reversely transcribed with Moloney murine leukemia virus (MMLV) reverse transcriptase (Invitrogen, USA) using random hexamers as primers. The resulting cDNAs were then subjected to qRT-PCR analyses.*β-ACTIN* (accession number: EU250003.1) was selected as reference gene. Primers were designed according to the* IbCAT2* and*β-ACTIN* sequence by using Primer Premier 5.0. qRT-PCR was performed using SsoFast EvaGreen Supermix (Bio-Rad, USA) on an iCycler MyiQ Real-Time PCR (Bio-Rad, USA) according to the manufacturer's instruction with IbCAT F2 (5′-CTGTGGGTCGCTTGGTTT-3′; bp position: 905–922), IbCAT R2 (5′-CAAGACGATGTCGCTGAGTAT-3′; bp position: 1049–1069), IbACT F1 (5′-GGTGTTATGGTTGGGATGGGAC-3′; bp position: 130–151), and IbACT R1 (5′-GGTAAGAAGGACAGGGTGCTC-3′; bp position: 304–324) as primers. The cycling conditions were as follows: 40 cycles of 2 min at 95°C, 10 s at 95°C, 15 s at 56°C, and 20 s at 72°C. The relative quantification results were then calculated by 2^−ΔΔCT^ method [33].

### 2.5. Overexpression of* IbCAT2* in* E. coli* and Stress Response Analysis

The plasmid pET32-CAT was transformed into the* E. coli *host strain Rosetta (DE3) and shook at 37°C for approximately 2 h in 2 mL LB medium with 50 *μ*g/mL ampicillin and 1 mM IPTG. After the incubation at 18°C for 16 h, sonication was used to release the target proteins. The supernatant was subjected to SDS-PAGE analysis after high speed centrifugation.

The recombinants expressing* IbCAT2* were selected and induced by 1 mM IPTG for 4 h. In salt stress analysis, cultures were diluted to 1 : 100 with 100 mL LB medium (containing 1 mM IPTG, 50 *μ*g/mL ampicillin, and 800 mM NaCl) and incubated at 37°C for 56 h. In drought stress analysis, cultures were diluted to 1 : 100 with 100 mL LB medium (containing 1 mM IPTG, 50 *μ*g/mL ampicillin, and 30% PEG6000) and incubated at 37°C for 72 h. The control cultures were diluted to 1 : 100 with 100 mL LB medium (containing 1 mM IPTG and 50 *μ*g/mL ampicillin) at 37°C for 24 h. OD_600_ was measured every 4 h during the incubation.* E. coli *with pET-32a(+) empty vector was used as a control for all treatments.

### 2.6. In Vivo Functional Characterization in* Saccharomyces cerevisiae*

The* Kpn* I/*Eco*R I fragment containing the cDNAs coding for the mature IbCAT2 peptide was cloned into the yeast expression vector pYES2 and then transformed into* S. cerevisiae* strain FGY217 (MATa, pep4Δ, ura3-52, and lys2Δ201) by LiAc/SS-DNA/PEG method [37] and selected on SD-URA plate (20 g/L agar, 1 mg/mL Lys, 1.7 g/L yeast nitrogen base without amino acids and ammonium sulfate, 5 g/L ammonium sulfate, and 20 g/L galactose). Verified single yeast colonies were firstly cultivated in liquid SD-URA medium at 30°C for 48 h. The OD_600_ was measured and adjusted to 1.0 and then used for the following resistance assays. For salt resistance analysis, yeast cells were collected from 1 mL yeast culture and resuspended in 1 mL 5 mol/L NaCl and incubated at 4°C for 48 h. Following incubation, the culture was diluted 1000 times and 50 *μ*L spread on to solid SD-URA and incubated at 30°C until a single colony could be observed. The numbers of resultant colonies for each SD-URA plate were then counted. For drought tolerance analysis, yeast cells were collected and resuspended in 1 mL 4 mol/L sorbitol or 40% PEG6000 and then diluted and spread onto lipid SD-URA to count colonies. At least three replicates were used for stress resistance assays. Yeast strains hosting empty vector pYES2 were used as a negative control.

### 2.7. Catalase Enzyme Activity Assay

A single colony was cultured in LB liquid medium overnight at 37°C, following which 0.5 mL was diluted in 50 mL LB liquid medium for IPTG induction. The control was not induced by IPTG. The bacterial cell densities of the induced recombinants and control strains were adjusted to the same value (the OD value adjusted to 0.6) after 8 h by dilution in LB liquid medium. The method of stress treatment is consistent with that of growth curve as above. Cultures were collected at 40 h (the OD value adjusted to 0.5) and 60 h (the OD value adjusted to 0.15) after salt and drought stress treatment, respectively, and adjusted to the same value. After centrifugation the supernatant was discarded and the precipitates were diluted in 10 mL precooled PBS buffer solution (Ph = 7.4). The target proteins were released from the cells by sonication. Cellular debris was separated by high speed centrifugation and 100 *μ*L of supernatant was used for the catalase activity assay using the Catalase (CAT) Detection Kit of Nanjing Jiancheng Bioengineering Institute (NJBI) according to the manufacturer's instructions. One unit of CAT activity was defined as 1 mL bacterial solution consuming 1 *μ*mol H_2_O_2_ for 1 second.

Determination of CAT enzyme activity of leaves was taken from well-watered group, salt treatment group, and drought treatment group, respectively. Enzyme activity was carried out using the Catalase (CAT) Detection Kit of Nanjing Jiancheng Bioengineering Institute as above. The frozen leaves kept at −80°C were homogenized with 100 mM phosphate buffer saline (PBS, pH 7.4) at a 1 : 10 ratio (fresh weight of leaf sample/buffer volume). The crude homogenates were centrifuged at 4°C and the supernatant was used to determine the enzyme activities.

The enzymatic activities of CAT were determined by lysates from yeast cells resuspended in 5 mol/L NaCl or 4 mol/L sorbitol. Firstly, yeast cells were collected by centrifugation at 8,000 rpm for 10 min at 4°C, and the pellet was resuspended in PBS (pH 7.4). Secondly, the suspension (5 ml) was lysed by adding the same volume glass beads (0.5 mm diameter) for 2-3 min. Then, the suspension was sonicated for 10 min by ultrasonic probe to break the cells. Specific activities were calculated by use of the crude lysates.

## 3. Results and Discussion

### 3.1. Sequence Cloning and Characterization of* IbCAT2*

**Figure 1:**
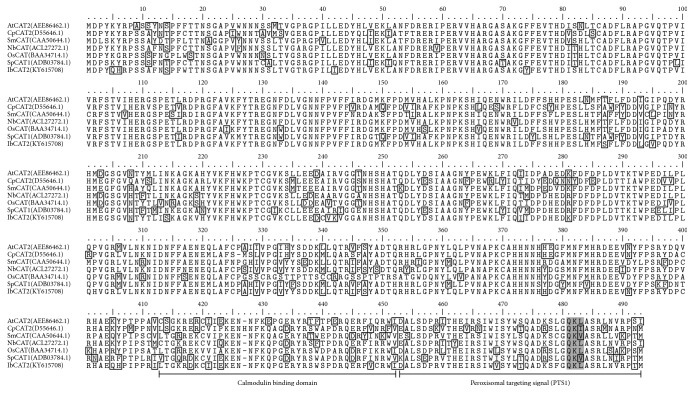
*The alignment of IbCAT2 with other plant catalases*. The boxes mean PTS1 (S-R-L) motif and Q-K-L motif. The underline indicates a putative calmodulin binding domain and a putative peroxisomal targeting signal domain. The shading section shows a classical motif (Q-K-L) of PTS1.

**Figure 2:**
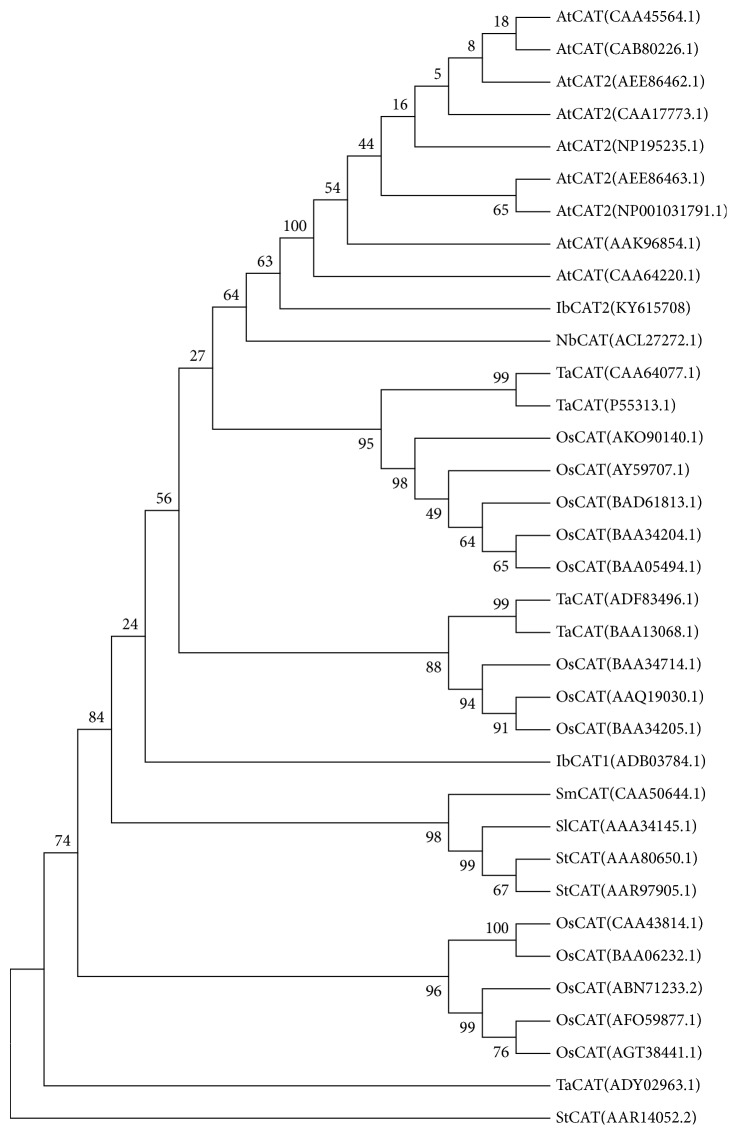
*Clustering analyses of IbCAT2 with CATs from other plant species*. CATs of some other plants were retrieved from Genbank and aligned by ClustalW [29] and Neighbor-Joining tree was constructed by MEGA6.0 [30].

Sweet potato can tolerate marginal growing conditions better than most of other crops (e.g., dry spells, poor soil). However, stress-resistant genes of this crop remain largely undetermined. Basing on the published comprehensive transcriptomes of sweet potato [32, 34], a full-length CAT encoding sequence was identified (named* IbCAT2*). Results showed that* IbCAT2* contains a 1479 nucleotides' (492 amino acids) open reading frame (ORF), which is the same as that of* SPCAT1* [26–28],* AtCAT1*,* AtCAT2*, and* AtCAT3* [24]. A sequence similarity search showed that IbCAT2 shared the highest identity with catalase isozyme 3 of* Nicotiana tabacum* (94%, gi∣1027858451) and catalase isozyme 3 of* Nicotiana tomentosiformis* (93%, gi∣697141344), and an identity of 77% with the published* SPCAT1 *[26–28]. These results suggest that* IbCAT2 *is different from* SPCAT1 *[26–28]. The subcellular localization of IbCAT2 was predicted by using Wolf psort [38]. Results showed that the kNN (*k*-nearest-neighbor) value of IbCAT2 was 14 (pero: 8, mito: 3, and chlo: 2), indicating that IbCAT2 should be located in peroxisomes. Comparison of several plant catalases suggested that the classical peroxisomal targeting signal 1 (PTS1) domain and calmodulin binding domain around the C-terminal region were also found in IbCAT2 (Figure 1). The PTS1 motif (Q-K-L), which has been shown to commonly exist in many CATs and directed catalase import into peroxisome by means of influence on the interaction between CAT and the PTS1 receptor protein Pex5p [29, 30], was identified at the extreme C-terminus of IbCAT2. SPCAT1 has been shown to be potentially regulated and activated by calmodulin and calcium [28]. Similarly, a putative calmodulin binding domain is located from the 415th Gly (G) to 451st Ile (I) in IbCAT2. It was demonstrated that calmodulin and calcium played an important role in posttranslational regulation of catalase in* Arabidopsis* and sweet potato [31]. Similarly, a putative calmodulin binding domain is located from the 415th Gly (G) to 451st Ile (I) in IbCAT2. Known CATs from other plant species were retrieved from Genbank (https://www.ncbi.nlm.nih.gov/) and aligned by ClustalW [39], after which a Neighbor-Joining tree was constructed by MEGA6.0 [40]. Results showed that IbCAT2 can be clustered with several well-characterized CATs (Figure 2). These results prove that the cloned I*bCAT2* encodes a putative peroxisomal catalase, which is likely regulated and activated by calmodulin and calcium.

### 3.2. Spatial Expression Patterns of* IbCAT2*

**Figure 3:**
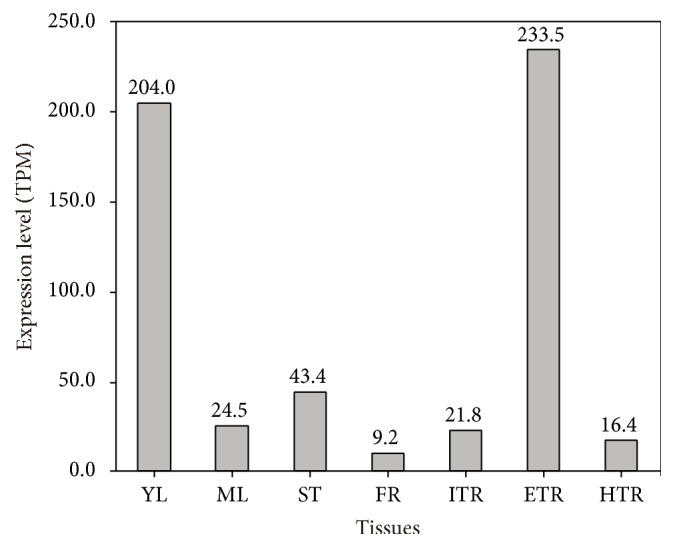
*Spatial expression pattern of IbCAT2*. DGE tags of seven samples were aligned to* IbCAT2* using Bowtie (v2.0.0-beta5) [31]. The number of mapped tags from each sample was normalized by the TPM algorithm [28, 32]. YL: young leaves; ML: mature leaves; ST: stems; FR: fibrous roots; ITR: initial tuberous roots; ETR: expanding tuberous roots; HTR: harvest tuberous roots.

Since its inception, digital gene expression (DGE) profiling has been a widely used method for global expression profiling. In a previous study, seven different sweet potato samples were collected for DGE analyses [32]. DGE tags from these samples were reused to align to* IbCAT2* using Bowtie (v2.0.0-beta5) [41]. The mapped tag number of each sample was normalized by TPM algorithm (number of transcripts per million clean tags) [35, 36]. Results showed that* IbCAT2* was highly expressed in young leaves (204.0 TPM) and expanding tuberous roots (233.5 TPM), while the expression levels in other tissues were all lower than 50 TPM (Figure 3). Fibrous roots had the lowest abundance (9.2 TPM).

### 3.3. Stress Response of* IbCAT2* in Sweet Potato

**Figure 4:**
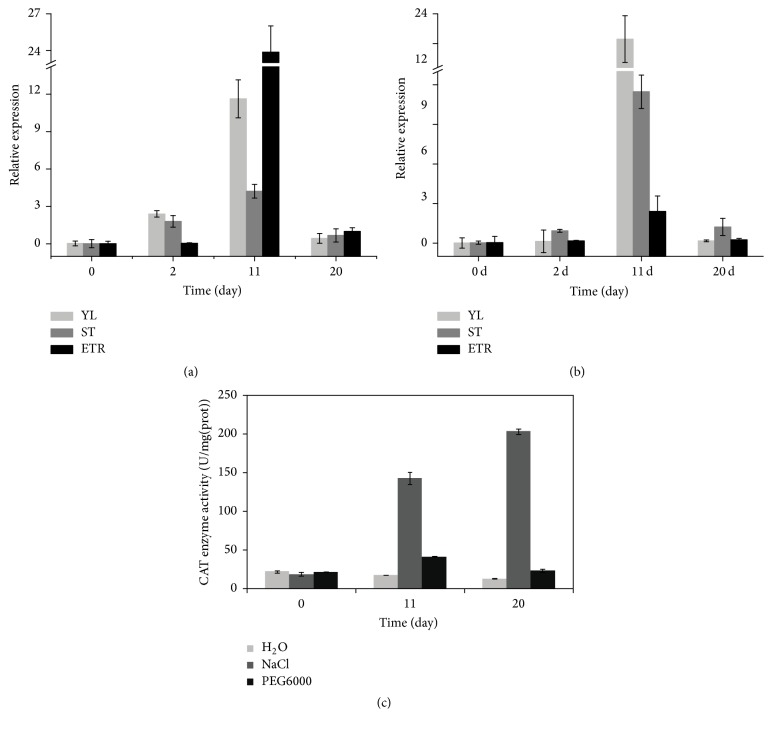
*Expression patterns and enzyme activity assay of IbCAT2 in stress-treated sweet potato*. Sweet potato plants were planted in the glasshouse under natural irradiance conditions for three months and then treated with 200 mmol/L NaCl (a) and 10% PEG6000 (b). Young leaves (YL), stems (ST), and expanding tuberous roots (ETR) with or without stress treatment were collected for expression analysis of* IbCAT2*. *β-ACTIN* was used as an internal control. The quantification results were calculated by 2^−ΔΔCT^ method using the plant materials without stress treatment as control [33]. Total CAT enzyme activity assay of* IbCAT2* (c) in stress-treated leaves of sweet potato.

To analyze the stress response of* IbCAT2 *in sweet potato cv. Xushu 18, plants were irrigated with 10% PEG6000 or 200 mmol/L NaCl solutions. Young leaves (YL), stems (ST), and expanding tuberous roots (ETR) with or without stress treatment were collected at 0, 2, 11, and 20 days after treatment for qRT-PCR analyses at 9:00 AM. The relative quantification results were calculated by 2^−ΔΔCT^ method using the plant materials without stress treatment as control [33]. Before starting stress treatment,* IbCAT2* had a relatively low expression level (0.01 to 0.04) when compared with that of*β-ACTIN* (1.00). After being exposed to 200 mmol/L NaCl, the level of* IbCAT2* in YL was upregulated 72.74 and 352.48 times compared with that in the control (under natural conditions) at 2 days and 11 days, respectively, but the expression abundance decreased after 11 days (Figure 4(a)). IbCAT2 expression in ST was also upregulated at 2 days (1.80) and persistently increased to 11 days (4.22) and then decreased to 0.68. In the ETR, IbCAT2 expression did not have a very sensible difference between 0 days and 2 days but increased greatly and reached the highest levels at 11 days. After being exposed to 10% PEG6000, the expression of* IbCAT2* showed the same tendency as that of salt-treated sweet potato (Figure 4(b)).* IbCAT2* abundance increased by 9.73, 26.04, and 4.08 times at day 2 days in YL, ST, and ETR and was upregulated 1532.60, 318.66, and 51.15 times at day 11, respectively. However, the expression was downregulated after 11 days with expression levels of* IbCAT2* being 0.17, 1.23, and 0.25 in YL, ST, and ETR, respectively.

As an indicator of antioxidant capability in plants, catalase can detoxify ROS generated in stress responses. Numerous studies demonstrate that expression abundance of CATs can be noticeably upregulated at the beginning of stresses for the scavenging of ROS [30, 42, 43]. With time, plants will activate a nonenzymatic antioxidant system to decrease oxidative damage [44] and reduce the expression of catalases [30]. Thus, when exposed to 10% PEG6000 and 200 mmol/L NaCl,* IbCAT2* was initially upregulated and then decreased gradually after 11 days. CAT expression is usually affected by many factors, such as subcellular localization, some other antioxidant enzymes, and developmental stages [20]. Hence, the expression level and degree of expression change of* IbCAT2* varies in different tissues. In conclusion, the* IbCAT2* cloned in this study should be an important stress-resistant gene.

Drought and salt all induce the accumulation of ROS such as superoxide, hydrogen peroxide, and hydroxyl radicals [45]. Catalase is essential for the removal of H_2_O_2_ produced in the peroxisomes by photorespiration [46]. In the majority of cases, the expression level and enzyme activity of CAT under abiotic stress was higher than control [47, 48]. Total CAT enzyme activity of sweet potato leaves was determined as demonstrated in Figure 4(c). In general, CAT enzyme activity was upregulated under salt or drought condition and not changed in control. 200 mmol/L NaCl caused the highest enhancement of CAT activity which increased along with time. On the other hand, under 10% PEG6000, there are about 2.4 and 1.8 times increase in CAT activity.

The enzymatic activity and the gene expression level of leaves were examined, but the two are not identical. This result may flow from two causes: the first is that the CAT activity was not always accompanied with mRNA levels [49]; the second is that the CAT activity tested was total CAT, but the expression level contains only CAT2.

### 3.4. Overexpression of* IbCAT2* Conferred Salt and Drought Tolerance in* E. coli*

**Figure 5:**
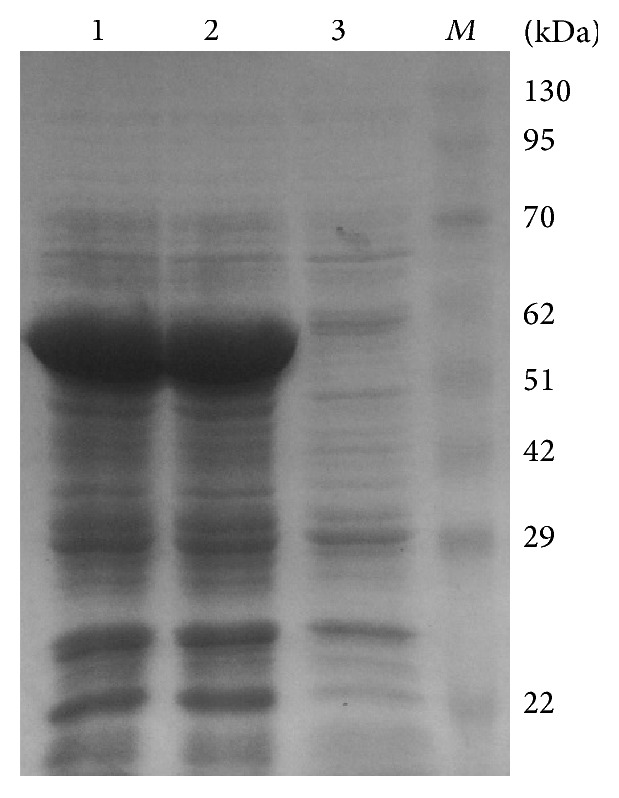
*Overexpression assay of IbCAT2 in E. coli*.* IbCAT2* was overexpressed in* E. coli* and examined by SDS-PAGE. SDS-PAGE analysis of IbCAT2 overexpression in* E. coli* BL21; Lane *M*, protein marker; lane 1 and lane 2, crude extracts of* E. coli* BL21 containing the pET32-CAT with IPTG (1 mM); lane 3, crude extracts of* E. coli* BL21 containing the pET32-CAT without IPTG induction.

To preliminarily characterize the function of* IbCAT2*, the recombinant plasmid (pET32-CAT) was transformed into* E. coli* host strain Rosetta (DE3) and used for stress tolerance analysis. Firstly, the recombinants were induced by 1 mM IPTG and total proteins separated by SDS-PAGE (Figure 5). Results showed that a clearly distinct protein band with molecular size between 51 kD and 62 kD existed in the supernatant, which correlated with the deduced protein size (57.04 kD). This result indicated that IbCAT2 was expressed as soluble protein in* E. coli* and the culture conditions were used for the following analyses.

**Figure 6:**
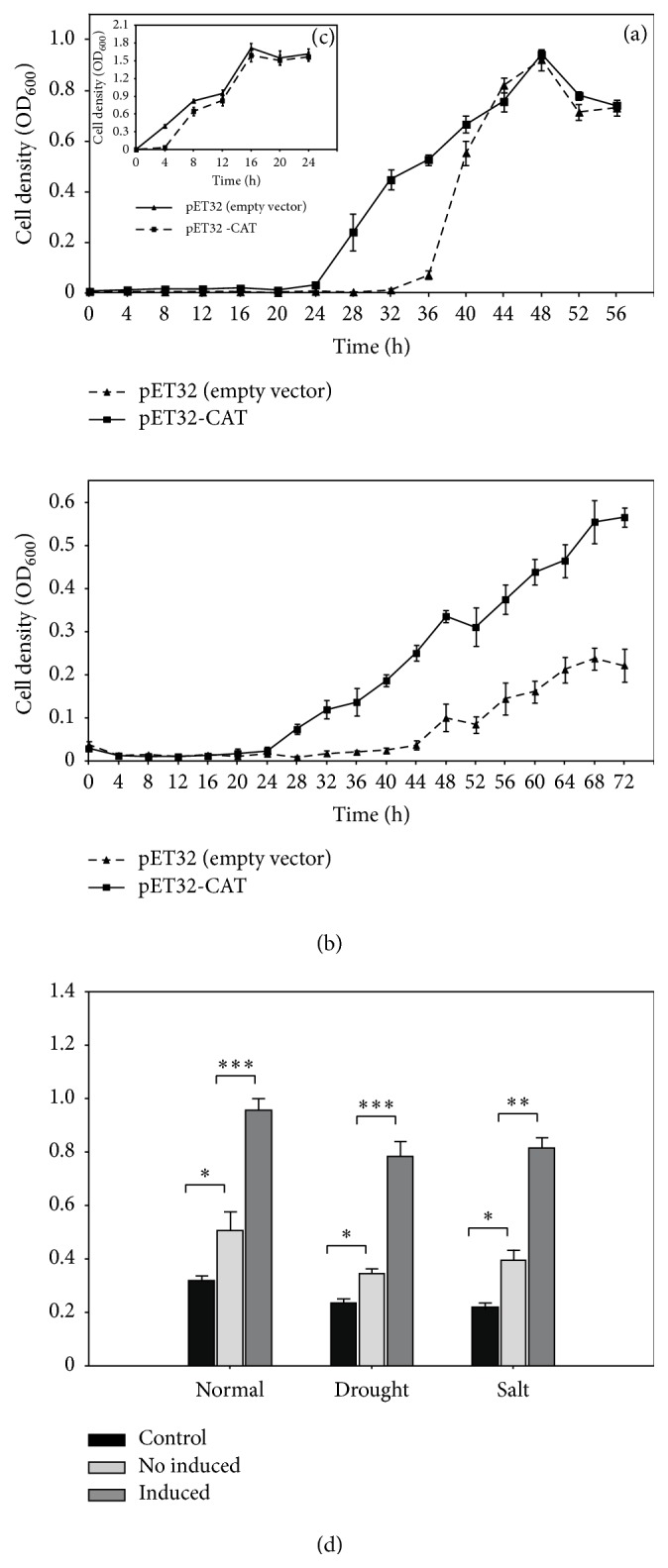
*Salt and drought tolerance analysis of IbCAT2 in E. coli*. Growth curve of recombinants and control strains exposed to salt stress (a), drought stress (b), and normal LB medium (c). Enzyme activity was assayed by determination kit of Nanjing Jiancheng Bioengineering Institute NJBI (d). ^*∗*^*p* ≤ 0.05, ^*∗∗*^*p* ≤ 0.01, and ^*∗∗∗*^*p* ≤ 0.001.

In normal LB medium, the control strain (hosting empty vector) had a shorter period of adjustment and a faster growth rate than the recombinants (Figures 6(a) and 6(c)), which is due to the enhanced transcription and translation in* E. coil* due to the overexpression of* IbCAT2*. As more amino acids were used for the biosynthesis of IbCAT2, the basal metabolism was negatively impacted in the recombinants. For stress analysis, recombinants were firstly induced by IPTG for 4 h and then diluted and exposed to 800 mM NaCl or 30% PEG6000. For salt stress analysis, recombinants and the control strain were grown in LB liquid medium (with 800 mM NaCl) at 37°C for 56 h and OD_600_ measured each four hours (Figure 6(a)). The recombinants barely grew during the first 24 h but then grew rapidly in the following 24 h until OD_600_ ≈ 0.94. The control strain, which hosted an empty vector, barely grew in the first 32 h but grew quickly after 36 h. Following being exposed to 30% PEG6000, the growth of the* E. coli* strains was almost completely suppressed in the first 24 h (Figure 6(b)). The recombinants exhibited a fast period of growth during 24–72 h and the OD_600_ reached 0.57 at 72 h, while the control strain began its growth from 44 h and the OD_600_ at 72 h was 0.22.

In order to confirm whether the protein product actually has catalase activity, enzymatic activity was assayed using the determination kit of Nanjing Jiancheng Bioengineering Institute (NJBI) according to the manufacturer's instructions. Results showed that the catalase activity can be determined in* E. coli* recombinants and the control strain hosted empty vector (Figure 6(d)). Enzymatic activity determined in the control strain could be derived from the endogenic catalase in* E. coli*. However, the recombinants demonstrated much higher catalase activity, and IPTG induction significantly increased the activities. Under normal growth condition, the catalase activity of the control strain was 0.32 U/mL bacteria solution, while those for drought and salt-treated strains were 0.23 and 0.22 U/mL. The activities of IPTG induced recombinants were 0.95, 0.78, and 0.82 U/mL for normal, drought, and salt growth conditions, respectively, which were significantly higher than that of “no induced.”

### 3.5. *IbCAT2* Overexpression in* S. cerevisiae* Resulted in Salt and Drought Tolerance

**Figure 7:**
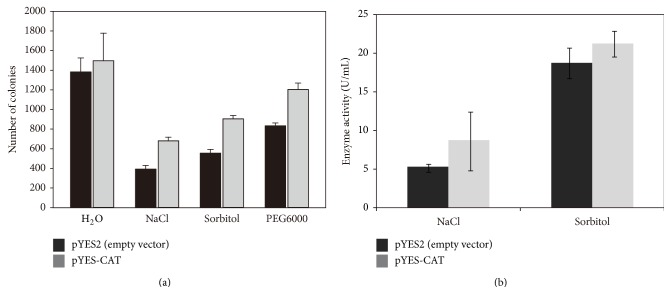
*Salinity and drought tolerance analysis of IbCAT2 in yeast*. The surviving colony numbers of yeast under drought or salt stress (a); changes of enzymatic activities of CAT between control and recombinant strain during salt or drought treatment (b).

As an eukaryote,* S. cerevisiae* has been developed as an important expression system. To confirm the stress tolerance observed in* E. coli*, the coding sequence of IbCAT2 was cloned and expressed in* S. cerevisiae*. When recombinants were exposed to salt and drought stress, results showed that the surviving colony numbers of* IbCAT2* overexpressing yeast were significantly higher than that of the control (hosting empty vector) (Figure 7(a)). Once treated with 5 mol/L NaCl, a total of 681.3 ± 37.2 colonies grew on SD-URA plates, while that of the control was 393.3 ± 36.3. Following being exposed to 4 mol/L sorbitol or 40% PEG6000, 904.0 ± 34.2 and 1204.0 ± 44.1* IbCAT2 *containing colonies survived, respectively, but only 554.7 ± 39.5 and 833.3 ± 30.6 colonies for the control strains, respectively, a significantly lower proportion.

The activities of CAT in the recombinant strain (containing pYES-CAT) and the control (containing pYES2) treated with NaCl or sorbitol are presented in Figure 7(b). As shown in Figure 7(b), the recombinant cells had better catalase activities than the control strain under drought or salt stress. Under NaCl stress, the increase of CAT activities in transgenic strain was about 1.6-fold that of control after treatment. Although increased activity of CAT was only about 13.3% under sorbitol, it showed higher catalase activities than strains under salt stress. It was shown that the activity of CAT enzyme was in agreement with the surviving colony numbers of yeast under drought or salt stress. These results further confirmed that the increased CAT activities were positively related to stress resistance in yeast.

## 4. Conclusions

In conclusion, a peroxisomal catalase encoding gene,* IbCAT2*, was identified and cloned from sweet potato cv. Xushu 18. IbCAT2 was shown to increase tolerance to abiotic stresses in* E. coli* and yeast cells. Enzymatic activity also proved that the induced recombinants with* IbCAT2* had a higher catalase activity than that of “no induced” or strains with empty vector under abiotic stresses.* IbCAT2* expression was affected by drought or salt treatment in sweet potato, and the expression level and degree of expression change of* IbCAT2* vary in different tissues. The positive response of* IbCAT2* to abiotic stresses suggested that* IbCAT2* may play an important role in stress responses.
